# A Critical Role for *Toxoplasma gondii* Vacuolar Protein Sorting VPS9 in Secretory Organelle Biogenesis and Host Infection

**DOI:** 10.1038/srep38842

**Published:** 2016-12-14

**Authors:** Takaya Sakura, Fabien Sindikubwabo, Lena K. Oesterlin, Hugo Bousquet, Christian Slomianny, Mohamed-Ali Hakimi, Gordon Langsley, Stanislas Tomavo

**Affiliations:** 1 Laboratory of Cellular and Molecular Biology of Toxoplasma, Université de Lille, Institut Pasteur de Lille, Center for Infection and Immunity of Lille, INSERM U 1019, CNRS UMR 8204, 59000, Lille, France; 2 Institute for Advanced Biosciences (IAB), INSERM U1209, CNRS UMR5309, Université Grenoble Alpes, 38700, Grenoble, France; 3 Institut Curie, PSL Research University, CNRS UMR144, Molecular Mechanisms of Intracellular Transport Group, 75248, Paris, France; 4 Laboratory of Cell Physiology, INSERM U 1003, Université de Lille, 59655, Villeneuve d’Ascq, France; 5 Laboratoire de Biologie Cellulaire Comparative des Apicomplexes, INSERM U1016, CNRS UMR8104, Institut Cochin, 75014, Paris, France

## Abstract

Accurate sorting of proteins to the three types of parasite-specific secretory organelles namely rhoptry, microneme and dense granule in *Toxoplasma gondii* is crucial for successful host cell invasion by this obligate intracellular parasite. Despite its tiny body architecture and limited trafficking machinery, *T. gondii* relies heavily on transport of vesicles containing proteins, lipids and important virulence-like factors that are delivered to these secretory organelles. However, our understanding on how trafficking of vesicles operates in the parasite is still limited. Here, we show that the *T. gondii* vacuolar protein sorting 9 (*Tg*Vps9), has guanine nucleotide exchange factor (GEF) activity towards Rab5a and is crucial for sorting of proteins destined to secretory organelles. Our results illuminate features of *Tg*Vps9 protein as a key trafficking facilitator that regulates protein maturation, secretory organelle formation and secretion, thereby ensuring a primary role in host infection by *T. gondii*.

*Toxoplasma gondii* is an important food and waterborne pathogen causing toxoplasmosis, a usually mild disease in immunocompetent humans that can turn into a major threat in immunocompromised patients and during primary infection of pregnant woman. *T. gondii* is a member of the *Apicomplexa*, a phylum of numerous medically important parasites causing life-threatening diseases in human and animals worldwide. The phylum is typified by specific secretory organelles called rhoptries, micronemes and dense granules that are essential for host cell invasion and host pathway modulation. In *Toxoplasma,* rhoptries contain two groups, termed rhoptry (ROP) and rhoptry neck (RON), of effector proteins some of which are virulence factors; whereas micronemes secrete MIC proteins that are involved in parasite gliding, host cell attachment and invasion^1^,^2^. After invasion, dense granules discharge GRA proteins involved in parasitophorous vacuole (PV) formation and in hijacking host cell gene expression and metabolism^3^.

Despite having a single cell architecture, the parasite relies on active and abundant vesicle and protein trafficking. *T. gondii* and likely all *Apicomplexa* have reutilized classical endosomal and endocytic trafficking pathways more typical of higher eukaryotes towards building specialized secretory organelles that release parasite effectors to interplay with host cell signaling pathways as a way to take control over host immunity and ultimately to promote long-term parasitism^4^,^5^,^6^,^7^,^8^. It is now well established that apicomplexan parasites operate an unconventional endosome-like system (ELC) to traffic proteins from the Golgi apparatus to rhoptries and micronemes^6^,^7^,^8^. However, the mechanisms involved in endosome-like vesicle formation and delivery to the aforementioned organelles in general remain elusive. In mammalian cells, the endosomal system is used for the uptake of plasma membrane-associated components, which after passage through Rab5-positive early endosomes (EE) enter either Rab11A-positive recycling endosomes to return to the plasma membrane, or Rab7-positive late endosomes to be delivered to lysosomes (LE)^9^.

Clearly, regulated vesicular traffic allows different cargoes to correctly reach their specific organelle destinations at the right time^4^,^5^,^6^,^7^,^8^, and this is essential for successful parasite infection of its host^7^,^10^. For example, dynamin-related protein B (DrpB) and clathrin, which reside in the post-Golgi network (TGN) and the endosomal-like compartment (ELC) contribute to the formation of transport vesicles that are essential for secretory organelle biogenesis^11^,^12^. Vacuolar protein sorting 11 (Vps11) that is the subunit of CORVET (class C core vacuole/endosome tethering) and HOPS (homotypic fusion and vacuolar protein sorting) complexes are required for transport of MIC and ROP proteins to micronemes and rhoptries of *T. gondii*^13^. Thus, the parasite intra-vesicular trafficking of the endolysosome pathway involves functions of the CORVET and HOPS tethering complex. In addition, *T. gondii* Rab5+ and Rab7+ effector complexes likely interact with CORVET and HOPS in a manner similar to mammalian cells to induce membrane fusion within the endolysosome pathway of the parasite^14^,^15^.

We have described that transport of MIC and ROP proteins to microneme and rhoptry organelles, respectively, also required an essential sortilin-like receptor named *Tg*SORTLR^10^ and traffic through a non-conventional ELC^7^. The C-terminal tail of *Tg*SORTLR interacts with clathrin, three components of the AP1 adapter complex, Sec23/24 and three vacuolar protein sorting namely Vps26, Vps35 and Vps9^10^. Furthermore, the retromer composed of Vps35-Vps29-Vps26 that recycles *Tg*SORTLR from *Tg*Rab5- to *Tg*Rab7-dependent ELC before delivery to Golgi, is also essential for secretory organelle biogenesis and parasite shape^7^.

Here, we report that the *T. gondii* counterpart of Vps9 (herein named *Tg*Vps9) is a *bona fide* Rab5 GTP-Exchange Factor (GEF) that is crucial for ROP protein maturation and processing, and its loss leads to a reduced number of rhoptries. Absence of *Tg*Vps9 also impairs peripheral microneme biogenesis and disturbs dense granule secretion resulting in an accumulation of novel vesicles present both within and outside the parasite. Together with the rhoptry defect, absence of peripheral microneme formation and dense granule secretion severely affects parasite invasion of host cells. Collectively, these observations support the notion that *Tg*Vps9-mediated loading of GTP to *Tg*Rab5 is crucial for fine-tuning vesicle sorting to secretory organelles, the latter being essential for *T. gondii* host cell infection.

## Results

### *T. gondii* vacuolar protein sorting 9 is a *bona fide* Rab5 guanine nucleotide exchange factor

**Figure 1:**
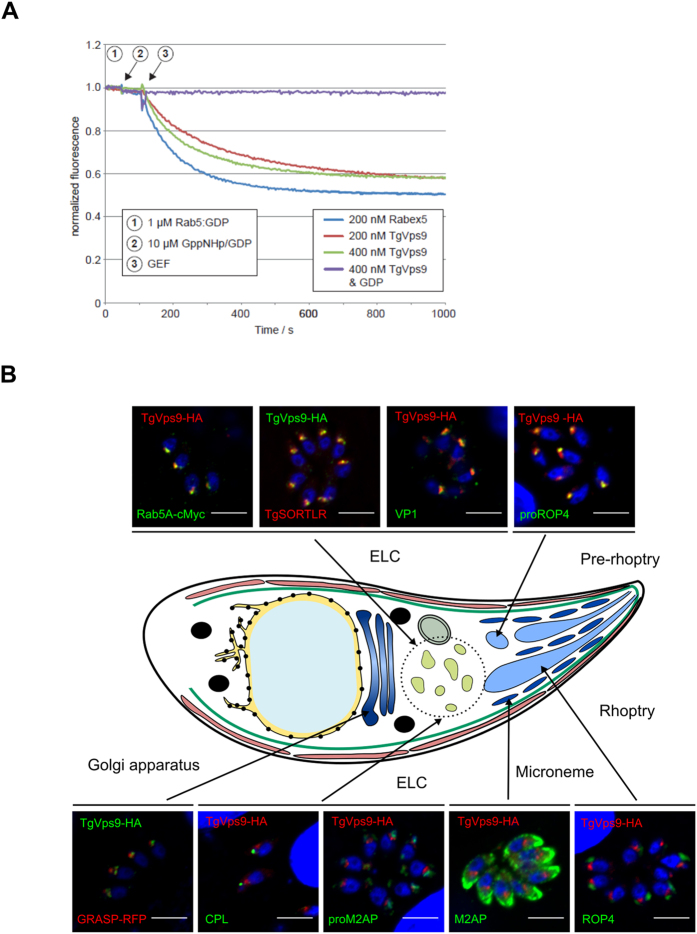
*T. gondii* Vps9 is a *bona fide* Rab5 GEF that localizes to endosome-like compartment. (**A**) *In vitro* GEF assay of recombinant *Tg*Vps9. The conformation changes of Rab5 upon GDP/GppNHp exchange were measured by monitoring tryptophan auto-fluorescence in the presence of 200 and 400 nM of *Tg*Vps9, respectively. As a positive control, the human Rabex5 known for its GEF activity to Rab5 was also tested. In the presence of GDP, no change in auto-fluorescence was observed either for *Tg*Vps9 or Rabex5 as no conformational change should be induced when exchanging GDP for GDP. (**B**) Subcellular location of *Tg*Vps9-HA in the knocked in parasites was compared to that of several other organelle markers such as TgRab5A-cMyc (early endosome marker), *Tg*SORTLR (Golgi and ELC marker), VP1 (Plant-like vacuole marker), pro-ROP4 (pre-rhoptry marker), GRASP-RFP (Golgi marker), CPL (Lysosomal-related compartment marker), pro-M2AP (immature microneme marker), and ROP4 (Rhoptry marker). Plasmids expressing GRASP-RFP and Rab5-cMyc were transiently transfected and RFP-positive parasites were directly visualized while specific anti-cMyc antibodies were used to stain Rab5-cMyc positive parasites. Specific antibodies were used to detect all other proteins. Scale bar indicates 5 μm.

In eukaryotic cells, Vps9 domain-containing proteins are known as guanine nucleotide exchange factors (GEF) that stimulate the release of monomeric guanosine diphosphate (GDP)-bound to Rab5, allowing guanosine triphosphate (GTP) to bind and activate Rab5 that, in turn, regulates endosome vesicle trafficking^16^,^17^,^18^. Previous work led us to identify an association with the C-terminus of *Tg*SORTLR^10^, a protein with a predicted molecular mass of 140 kDa typified by a Vps9-like domain localized between amino acid (aa) 945 and 1117. This putative parasite Vps9 homologue harbors a region of 1326 amino acids extended at the N-terminal end and in this respect differs from its yeast and human counterparts that contain a shorter N-terminal end (Supplementary Fig. S1A). We first demonstrated that the predicted *Tg*Vps9 domain operates *in vitro* as a GEF towards Rab5, by testing activity of a bacterial expressed *Tg*Vps9 recombinant protein towards human Rab5, as previously described^19^,^20^. Based on its homology with the catalytic core of mammalian Rabex5^21^; a truncated recombinant version (aa_849_ to aa_1134_) of *Tg*Vps9 was purified from *E.coli*. Human recombinant Rab5A was purified in its GDP bound form and nucleotide exchange to GppNHp, a non-hydrolysable GTP analogue, was monitored by tryptophan fluorescence measurements. A dose dependent GEF activity of recombinant *Tg*Vps9 towards human recombinant Rab5A was detected and compared to human recombinant Rabex5 (Fig. 1A). As expected, no tryptophan fluorescence change was observed in the presence of excess GDP as no conformational change was induced during nucleotide exchange from GDP to GDP (Fig. 1A).

### *Tg*Vps9 localizes to the endosome-like compartment of *T. gondii*

Having established that recombinant *Tg*Vps9 possesses GEF activity towards recombinant Rab5, we then sought to determine in *T. gondii* whether the protein resides in the same subcellular compartment as Rab5. To this end, we chromosomally appended the hemagglutinin (HA) epitope at the 3′-end of the endogenous *TgVps9* gene and validated by western blot that the tagged *Tg*Vps9 was readily expressed by transgenic tachyzoites (Supplementary Fig. S1B). HA-tagged *Tg*Vps9 migrated with an apparent molecular mass of 170 kDa, which is higher than the predicted 140.0 kDa, likely due to the observation that *Tg*Vps9 is heavily phosphorylated with 30 different phospho-sites indicated in Supplementary Figure S1A and collated at ToxoDB (www.toxodb.org).

Consistent with its *in vitro* GEF activity towards Rab5^22^,^23^, *Tg*Vps9-HA *in vivo* co-localized with *Tg*Rab5A (Fig. 1B, top and left panel). As expected, we confirmed a co-localization between *Tg*Vps9 and *Tg*SORTLR (Fig. 1B, top panel), the endosomal-like compartment (ELC) receptor that has been used to efficiently pull down *Tg*Vps9^10^. The unprocessed precursor pro-ROP4 (Fig. 1B, top panel) known to be present in the ELC also co-distributes with *Tg*Vps9, while surprisingly proM2AP, a microneme marker, does not (bottom panel). In addition, VP1 a marker of the plant-like vacuole that is present in close vicinity to ELC co-distributes with *Tg*Vps9 (Fig. 1B, top panel). In contrast, CPL (lysosomal-related compartment marker), GRASP (Golgi reassembly stacking protein), M2AP (MIC2-associated protein) or ROP4 (rhoptry marker) do not co-distribute with *Tg*Vps9 (bottom panel). Taken together, these co-localization studies indicate that *Tg*Vps9 is embedded in the endosomal-like compartment (ELC) together with Rab5, a compartment with an established role in the formation of secretory organelles of *T. gondii*^4^,^5^,^6^,^7^,^8^,^10^.

### Conditional ablation of *TgVps9* affects secretory organelle biogenesis to generate large novel vesicular-like structures

**Figure 2:**
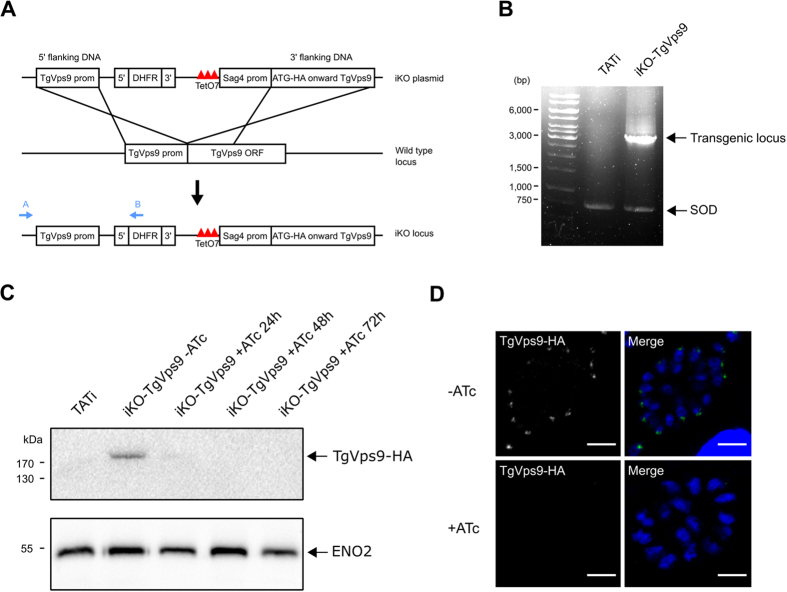
Conditional disruption of *Tg*Vps9 gene. (**A**) Schematic representation of the ATc-inducible strategy to generate a knock out *Tg*Vps9 mutant after a double homologous recombination in *T. gondii* genome. (**B**) PCR analysis to demonstrate the perfect integration of the knock-out vector into *Tg*Vps9 locus using two specific primers A and B indicated in blue color. PCR of superoxide dismutase (SOD) gene corresponds to a positive control indicating that equal quantity of genomic DNA was used for all parasite strains tested. (**C**) Western blots of total SDS protein extracts from wild type and iKO*Tg*Vps9 mutants, which were incubated without or with ATc for 24, 48, and 72 hours. Blots were probed with anti-HA antibodies but also with antibodies specific to the glycolytic enzyme ENO2 used as protein loading control. (**D**) Confocal images of ATc-untreated or ATc-treated iKO*Tg*Vps9 mutants that were stained with anti-HA antibodies at 48 h post-treatment and infection. Scale bar indicates 5 μm.

**Figure 3:**
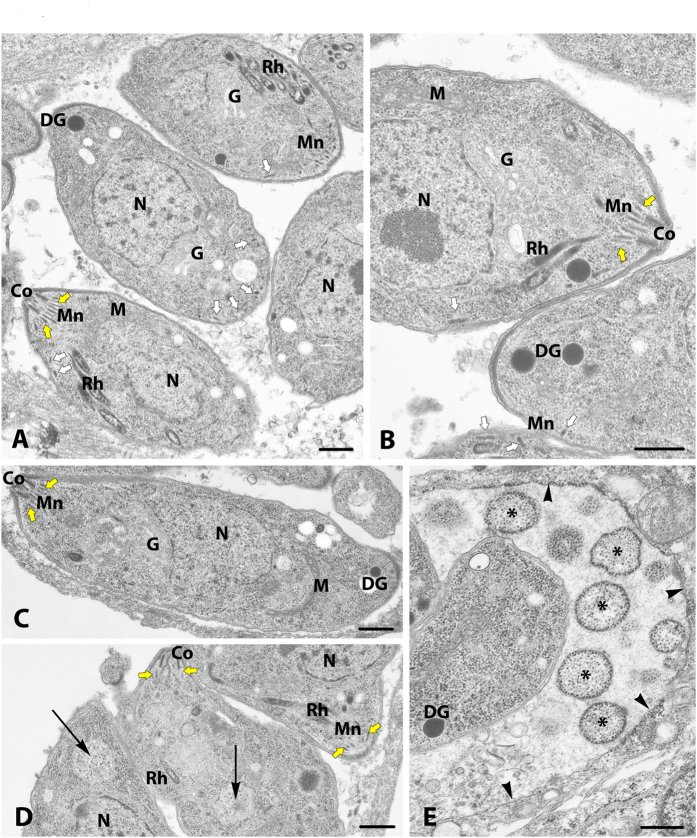
Electron microscopy of *Tg*Vps9 mutants. (**A**,**B**) Ultrastructural sections of typical parental parasites were shown. Secretory organelles such as rhoptries (Rh), both peripheral (Mn, white arrows) and apical micronemes (Mn, yellow arrows) and dense granules (DG) can easily be visualized. (**C**–**E**) Ultrastructural images of sections representing *Tg*Vps9-depleted mutants after 48 h of ATc treatment were shown. Only apical micronemes, few rhoptries and dense granules can be seen in *Tg*Vps9-depleted mutants. Examples of novel 500 nm-diameter of vesicles present in the cytoplasm above the nuclei of these mutants were indicted by arrows in panel D. A region of the parasitophorous vacuole (PV) space that also containing several novel vesicles of different sizes and morphology (see asterisks) were shown along with the delimited PV membrane (PVM) indicated by black arrowheads. The nucleus (N), mitochondrion (M), Golgi apparatus (G) and conoid (Co) were labelled. Scale bar is 500 nm.

To examine *Tg*Vps9 function in a clonal homogenous parasite population, we generated conditional anhydrotetracyclin (ATc)-inducible knockout *TgVvps9* mutants (named *iKOTgVps9*) using the strategy depicted in Fig. 2A. We selected several positive clones from the emerging stable parasite population and the genome editing of one expanded clone *in vitro* was verified by PCR using the two specific primers (see nucleotide sequences in Methods) shown in blue arrow (Fig. 2A), thus demonstrating the perfect integration of the knock-out vector at the *Tg*Vps9 locus (Fig. 2B). Following ATc treatment, while a significant reduction of HA-*Tg*Vps35 protein was observed 24 h post-treatment, 48 h or 72 h of ATc-treatments were required for a complete and reproducible disappearance of HA-*Tg*Vps35 protein by western blotting (Fig. 2C). We confirmed these latter observations by confocal imaging (Fig. 2D) and further investigated all phenotypic consequences of this ATc-inducible *Tg*Vps9 knock out mutant at least at 48 h post-treatment. Next, we examined iKO*Tg*Vps9 mutants by electron microscopy and observed several striking ultrastructural changes associated with the loss of *Tg*Vps9. Both apical (yellow arrows) and peripheral micronemes (white arrows) were observed in cytoplasm anterior to the nucleus of ATc-untreated tachyzoites (Fig. 3A,B) whereas ATc-treated iKO*Tg*Vps9 parasites, micronemes were only observed in the apical tip close to the conoid (Fig. 3C–E). We counted a total number of 30 micronemes located at the extreme apical end of 43 ATc-treated iKO-*Tg*Vps9 mutants using electron microscopy while a total number of 199 apical and peripheral micronemes were seen in 29 ATc-untreated parasites, indicating that there were about 5-fold fewer micronemes in *Tg*Vps9-deficient mutants *versus* the parental strain. Clearly, these data indicate the absence of peripheral micronemes in ATc-induced *iKOTgVps9* mutants and the presence in the cytoplasm at the proximity of the nucleus of a novel large vesicular structure of approximately 500-nm diameter (panel D, black arrows). Elevated numbers of novel vesicles of variable size and morphology were also observed in the PV space delimited by the PVM (panel E, shown with *). We estimated that about 17% of iKO*Tg*Vps9 mutants examined by electron microscopy contained these aforementioned 500-nm intra-parasite vesicles while approximately 20% of mutants had novel vesicles of variable size and morphology in their PV space. In addition, we observed a significant reduction of the overall number of rhoptries per mutants using electron microscopy. Specifically, ATc-treated iKO*Tg*Vps9 mutants contained less than two thirds the relative number of rhoptries per mutant compared to the parental line. Also, we observed a disorganized ultrastructural morphology with the marked absence of the typical banana-shaped bodies in several *Tg*Vps9-depleted mutants (Fig. 3D), in a manner similar to the retromer iKOTgVsp35 mutants^7^ whereas untreated iKO*Tg*Vps9 parasites appeared structurally normal with all secretory organelles (Fig. 3A,B). These latter observations suggest that the cytoskeleton of parasite bodies may also be affected in these mutants. It should be mentioned that electron microscopy was used to show that other organelles including the mitochondrion, the nucleus, the Golgi apparatus, the inner complex membrane (IMC) and the plasma membrane appeared morphologically normal in these iKO*Tg*Vps9 mutants treated with ATc for 48 h (Supplementary Fig. S2, see panel A–E). In addition, iKO*Tg*Vps9-deficient mutants appear to undergo normal endodyogeny with two daughters forming within the mother cell (Supplementary Fig. S2F, see stars indicating the nucleus of two dividing daughter tachyzoites). Collectively, these data suggest that the traffic to and the integrity of the other parasite organelles were not altered by the loss of *Tg*Vps9. Furthermore, rhoptries, micronemes and dense granules were not completely absent either or not morphologically affected *per se* in *Tg*Vps9-deficient mutants (Fig. 3 and Supplementary Fig. S2), only organelle number was reduced in these mutants. Taken together, these data indicate that *Tg*Vps9 likely regulates the turnover of vesicle precursors and pre-organelles destined to become fully mature secretory organelles.

### The loss of *Tg*Vps9 causes aberrant organelle secretion

**Figure 4:**
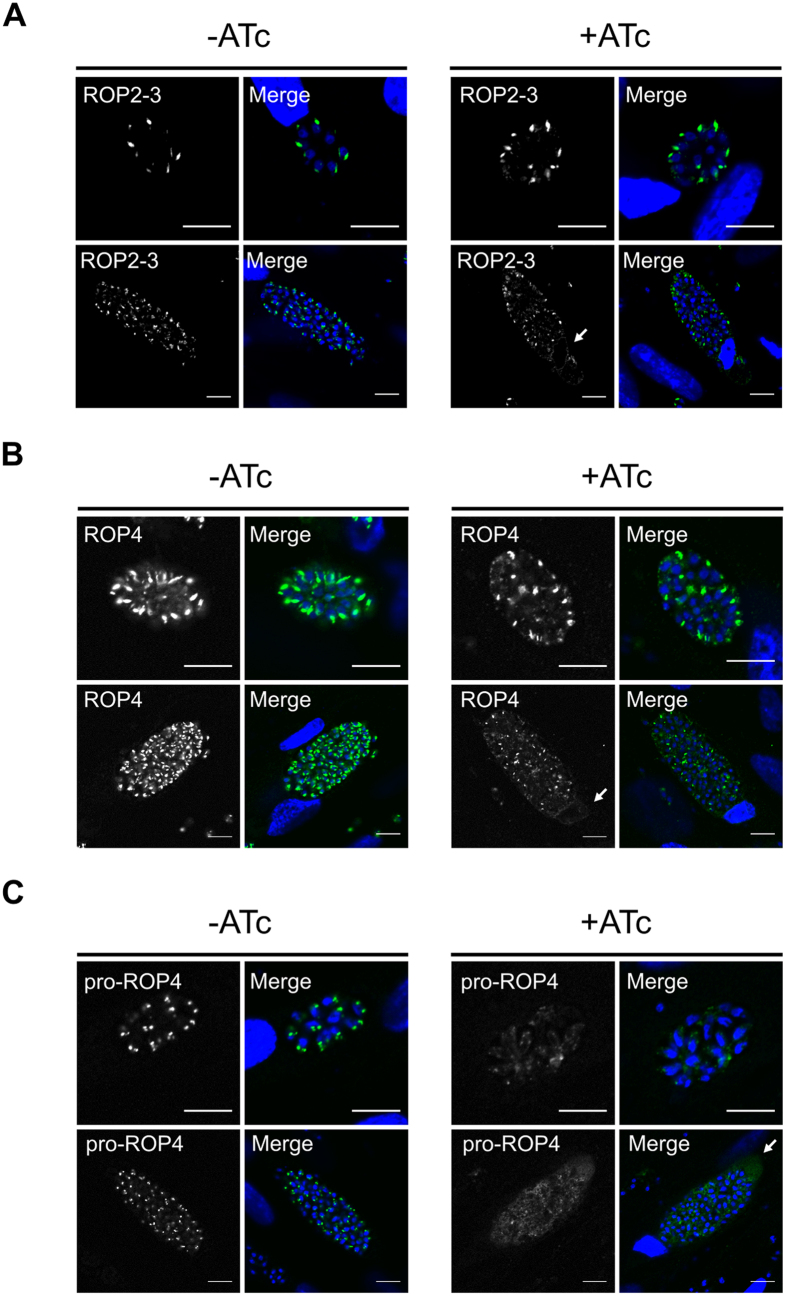
Conditional ablation of *Tg*vps9 results in mis-sorting of ROP proteins. (**A**) Confocal immunofluorescences of ROP2-3 proteins in iKO*Tg*Vps9 mutants in the presence (right panels) or absence of ATc (left panels) using specific antibodies to ROP2-3. (**B**) Confocal immunofluorescences of ROP4 proteins in iKO*Tg*Vps9 mutants in the presence (right panels) or absence of ATc (left panels) using specific antibodies to ROP4. (**C**) Confocal immunofluorescences of proROP4 proteins in iKO*Tg*Vps9 mutants in the presence (right panels) or absence of ATc (left panels) using specific antibodies to proROP4. Upper panels in (**A**–**C**) images correspond to small vacuoles containing 16 or less daughter parasites. Lower panels represent large vacuoles containing 32 or more daughter parasites. Scale bar on all images correspond to 10 μm.

We also examined the phenotypic consequences of *Tg*Vps9 loss on the subcellular localizations of different secretory organelle markers. In ATc-induced *iKOTgVps9* mutants, ROP2-3 and ROP4 proteins were abnormally sorted into the host cytoplasm and decorated the host cell nuclear envelope (Fig. 4A,B, right panels, see white arrows). In the absence of ATc, *iKOTgVps9* mutants displayed normal apical localization of ROP proteins (Fig. 4A,B, left panels). In ATc-induced *iKOTgVps9* parasites, pro-ROP4 was also profoundly mis-sorted (Fig. 4C, right panels) with diffuse and weak labeling in both the PV space and the host cell cytoplasm (white arrow) unlike non-ATc-induced parasites that showed the typical apical pre-rhoptry localization of pro-ROP4, i.e. proximal to parasite nuclei (Fig. 4C, left panels). The imaging data suggest that the iKOTgVps9 mutant-hosting PV may also be leaky following ATc induction for 48 h, thus resulting in the diffusion of pro-protein and mature protein in the vacuolar space and beyond the PV. Nevertheless, these data indicate that *Tg*Vps9 loss results in an accumulation of ROP precursor proteins, their mis-sorting to other subcellular compartments and a significant reduction of the number of rhoptries per parasites as observed by electron microscopy.

**Figure 5:**
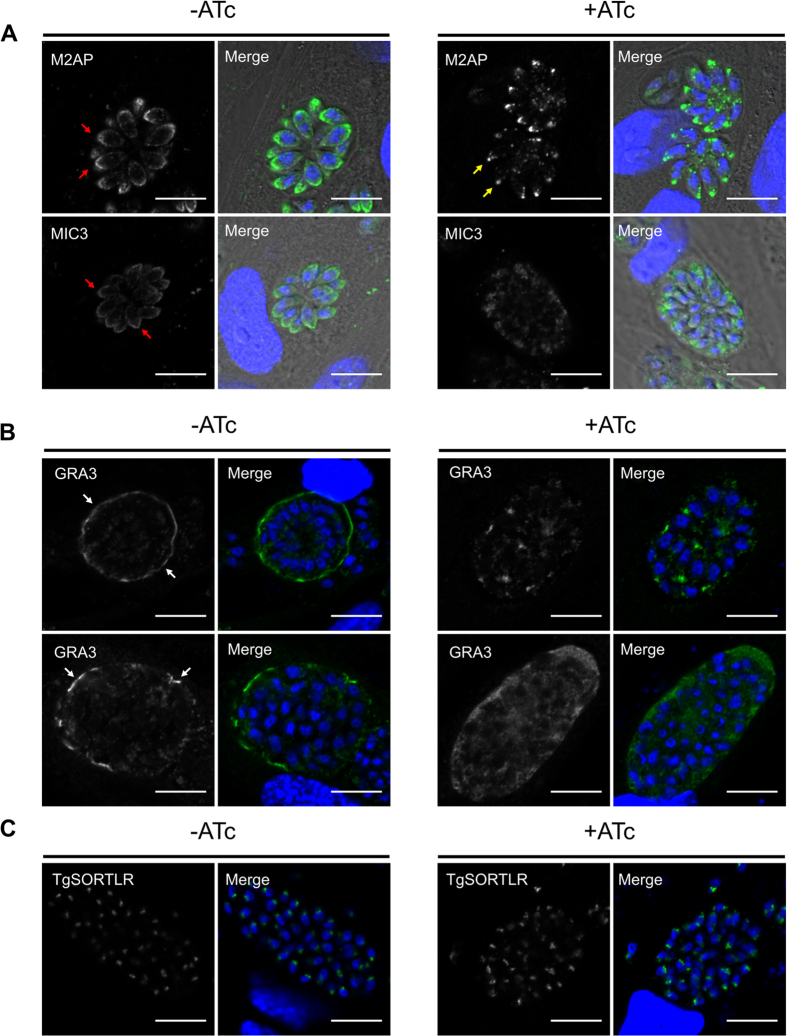
Conditional *Tgvps9* silencing affected microneme biogenesis and dense granule secretion. (**A**) Confocal immunofluorescences of M2AP and MIC3 proteins in iKO*Tg*Vps9 mutants in the presence (right panels) or absence of ATc (left panels) using antibodies specific to each protein, respectively. Note that the typical and conical signal of M2AP and MIC3 proteins (red arrows) in the parental strain disappears. Instead, only residual punctuated signal was seen in *Tg*Vps9-depleted mutants (yellow arrows), indicating the absence of peripheral micronemes in these mutants. The whole bodies of intracellular parasites were shown by phase contrast in order to indicate fluorescence signals corresponding to micronemes located at the extreme apical end of Cas9-GFP positive parasites (yellow arrows). (**B**) Confocal immunofluorescences of GRA3 protein in iKO*Tg*Vps9 mutants in the presence (right panels) or absence of ATc (left panels) using specific antibodies to GRA3. Note the absence of GRA3 protein delivery to the parasitophorous vacuole membrane (PVM) that contrasts to the situation in the parental parasites (white arrows). (**C**) The presence of *Tg*SORTLR in the Golgi-ELC region was unchanged in parental parasites and mutants regardless of treatment with ATc or not. Scale bar is 10 μm.

We confirmed in the conditional *Tg*Vps9 mutants that the typical conical microneme M2AP and MIC3 signals (Fig. 5A, left panels, red arrows) were completely changed to fluorescence signals at the extreme tip of each *Tg*Vps9-depleted mutant (Fig. 5A, right panels, yellow arrows). The most impressive and marked mis-sorting affects the dense granule GRA3 that was exclusively retained within the PV space (Fig. 5B, right panels) in the iKO*Tg*Vps9 mutants whereas this protein decorated the PV membrane of vacuoles containing parental parasites, as expected (Fig. 5B, left panels, white arrows). The location of *Tg*SORTLR was unchanged (Fig. 5C), confirming that not all proteins in the secretory ER-Golgi and ELC pathways are mis-sorted in *Tg*Vps9-deficient parasites. Altogether, these data clearly indicate that *Tg*Vps9 is required for correct protein trafficking, sorting and delivery to the three main secretory organelles: rhoptry, microneme and dense granules.

### Conditional *Tg*Vps9 silencing dysregulates ROP protein maturation

**Figure 6:**
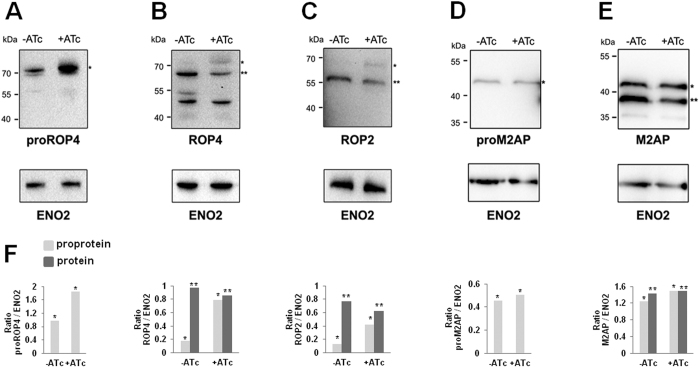
Upon conditional disruption of *TgVps9* unprocessed and immature ROP proteins accumulate. (**A**) Immunoblots of intracellular iKO*Tg*Vps9 mutants probed with specific anti-proROP4 at 48 h post-infection in the absence or presence of ATc. (**B**) Immunoblots of intracellular iKO*Tg*Vps9 mutants probed with specific anti-ROP4 antibodies at 48 h post-infection in the absence or presence of ATc. (**C**) Immunoblots of intracellular iKO*Tg*Vps9 mutants probed with specific anti-ROP2 antibodies at 48 h post-infection in the absence or presence of ATc. (**D**) Immunoblots of intracellular iKO*Tg*Vps9 mutants probed with specific anti-proM2AP antibodies at 48 h post-infection in the absence or presence of ATc. (**E**) Immunoblots of intracellular iKO*Tg*Vps9 mutants probed with specific anti-M2AP antibodies at 48 h post-infection in the absence or presence of ATc. (**F**) Quantification of protein intensity by densitometry that shows ratio between pro-protein or mature protein and ENO2 levels. The housekeeping glycolytic enzyme ENO2 was used as negative and loading control. A single star (*) indicates the unprocessed precursor protein while double stars (**) correspond to processed and mature protein. Molecular weights of protein markers (kDa) were indicated on the left of each panel.

In *T. gondii*, formation of rhoptries and micronemes is correlated with proteolytic processing followed by maturation of ROP and MIC proteins, respectively^24^,^25^,^26^,^27^,^28^. Therefore, we investigated the role of *Tg*Vps9 in the processing and maturation of representative ROP and MIC proteins. Specific antibodies that exclusively recognized the N-terminal pro-peptides of ROP4 revealed enhanced accumulation of pro-protein ROP4 in *Tg*Vps9-deficient mutants (Fig. 6A, left panel, single star). By calculating the ratio of the pro-protein to the mature ROP4 protein, using the housekeeping ENO2 loading control, we estimated that pro-ROP4 protein level was increased to almost 2-fold in ATc-treated iKO*Tg*Vsp9 mutants compared to untreated parental parasites (Fig. 6F). Using specific antibodies to the mature ROP4 protein, we detected a rise of proROP4 protein level to 4-fold (Fig. 6B, single stars), suggesting that pro-ROP4 protein accumulated between 2- to 4-fold higher in *Tg*Vps9-deficient mutants *versus* parental parasites (compare Fig. 6B,F, single star). As a consequence, the amount of processed mature ROP4 diminished (Fig. 6B,F, right lane, double stars). Similarly, we observed an increase level of unprocessed proROP2 (Fig. 6C,F, single star) that was estimated to be approximately 3-fold with a reduced amount of the mature ROP2 protein. We observed no significant changes for pro-M2AP protein (Fig. 6D–F, single star) or processed M2AP protein (Fig. 6E,F, double stars). We therefore concluded that pro-ROP proteins specifically accumulated in *iKOTgvps9* mutants, suggesting that *Tg*Vps9 is likely involved in the delivery of both proteases and pro-ROP proteins to the same subcellular compartment and that their proteolytic processing is important for rhoptry organelle formation.

### Conditional *Tg*Vps9 silencing abrogates host cell invasion by *T. gondii*

**Figure 7:**
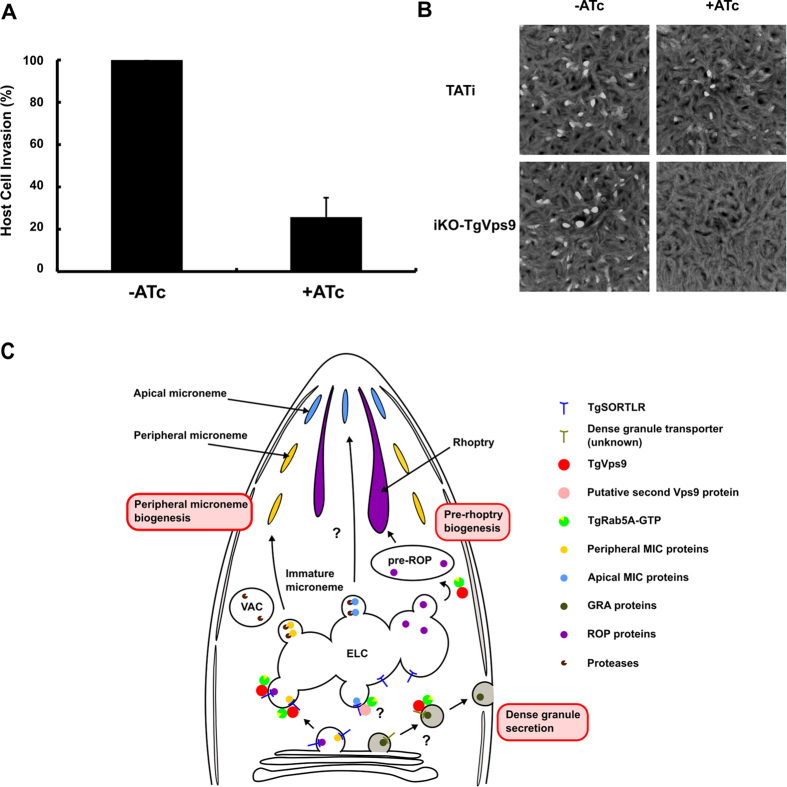
*Tg*Vps9 is essential for host cell invasion by *T. gondii*. (**A**) Host cell invasion was assayed in *iKOTgVps9* mutants in the presence or absence of ATc. Bars indicate mean ± SD (n = 3, P < 0.001 by Student’s test). (**B**) Host cell lytic plaques were examined in *Tg*Vps9-deficient mutants and parental RHΔ*Ku80*TATi parasites in the presence or absence of ATc. (**C**) Model of *Tg*Vps9-mediated trafficking and regulation of diverse sorting cargoes required for secretory organelle biogenesis in *T. gondii*. Based on the processing inhibition of precursor ROP proteins in *Tg*Vps9-delepted mutants and the mis-sorting of pro-ROP4, we conclude that the site of action of *Tg*Vps9 starts from the early endosome to late endosome until pre-rhoptry organelles. In addition, this also suggests that *Tg*Vps9 is required for the trafficking of the proteolytic enzymes to the pre-rhoptries where the pro-peptides of pro-ROP proteins were processed during protein maturation. In contrast, *Tg*Vps9 protein was not required to transport the cathepsin protease-like (CPL) enzyme^32^, a type II transmembrane protein involved in the processing and maturation of MIC proteins within the ELC. Indeed, *Tg*Vps9 function was limited to the biogenesis of peripheral micronemes. We further evidenced that *T*gVps9 protein was also important for the proper discharge of GRA protein inside the PVM. However, the molecular mechanisms underlying how GRA were transported to the dense granules and thereafter released to the PVM remain to be elucidated. We also propose that Rab5 is the key small GTPase, which was regulated by *Tg*Vps9 according to our biochemical GEF assays and the previous data of Vps11 of CORVET tethering complexes^13^ and those of Rab5A^33^. We suggest that function of *Tg*Vps9 is important for the transport to and secretion of organelles required for the successful intracellular lifestyle of the parasite.

More importantly, homogenous clonal populations of *iKOTgVps9* mutants allowed us to address the role of *Tg*Vps9 in *T. gondii* infection. The *iKOTgVps9* mutants were severely impaired in their ability to invade host cells with an 80% decrease after 48 h of ATc-treatment (Fig. 7A). In addition, at 7 day post-infection, *Tg*Vps9-depleted mutants did not form plaques associated with multiple rounds of host cell invasion in the presence of ATc (Fig. 7B), indicating that *Tg*Vps9 is essential for ensuring proper formation of secretory organelles that are necessary for parasite propagation through multiple cycles of invasion, lysis and reinvasion of host cells. We have not observed any obvious deficiency in parasite egress from the host cell as the ATc-treated iKO*Tg*Vps9 mutants spontaneously lysed out and freshly egressed tachyzoites can be recovered at 72 hours post-infection. Thus, we suggest that the reduction in number of secretory organelles *per* parasite and their default in organelle secretion represent the critical functions of *Tg*Vps9 that are required for proper host infection by *T. gondii*.

## Discussion

In this study, we established *in vitro* that *Tg*Vps9 is a *bona fide* Rab5 GEF and locates *in vivo* in the same compartment as *Tg*Rab5A. We are suggesting that the GEF activity of *Tg*Vps9 towards one of the *Tg*Rab5 isoforms is essential for host cell invasion by *T. gondii* and its intracellular propagation. Genetic ablation of *TgVps9* by ATc-inducible knock out system, led to impairment of microneme biogenesis and default in proper dense granule secretion. Overall, we observed a significant mis-sorting of rhoptry, microneme and dense granule proteins and we argue that this underpins the loss in host cell invasion, vacuole formation, PV leaking and intracellular propagation. We found an accumulation of unprocessed ROP proteins such as ROP2 and ROP4 following the loss of *Tg*Vps9. However, we did not observe pro-ROP4 in the endosomal-like compartment and the pre-organelles of *Tg*Vps9-deficient mutants. Instead, pro-ROP4 is released into the parasitophorous vacuole and the host cell cytoplasm, where together with other ROP proteins, it appears perinuclear. The common phenotypic traits of *Tg*Vps9 null mutants were reduced numbers of both rhoptries and micronemes *per* parasites, as seen by confocal imaging and electron microscopy. In contrast, we did not observe mis-sorting of proteins destined to mitochondrion, apicoplast, inner complex membrane and nucleus after *Tg*Vps9 loss using confocal imaging. No other morphological changes of the aforementioned compartments have been detected by electron microscopy. This suggests that the default in secretory organelles such as rhoptry, microneme and dense granule is specifically restricted to the function of *Tg*Vps9, as expected for a genuine partner of TgSORTLR^10^, the Golgi and endosomal-like receptor that has been previously shown to be involved in protein transport and biogenesis of these parasite-specific secretory organelles.

Based on these observations, we propose a model in which *Tg*Vps9 contributes to the regulation of ROP protein processing/maturation and the proper protein sorting to pre-rhoptries (Fig. 7C). This model is fully supported by the observation that the processing and maturation of ROP proteins have been shown to take place in the pre-organelles^1^ and pro-ROP4 co-localizes with *Tg*Vps9. In contrast, neither the pro-MIC2 associated protein (pro-M2AP), nor the pro-M2AP processing enzyme CPL^24^,^25^,^26^,^27^,^28^ colocalize with *Tg*Vps9. This suggests that both processing and maturation of MIC proteins likely occurs in the distal sub-compartment of the ELC^29^,^30^ by means of CPL and in a *Tg*Vps9-independent fashion. It is also tempting to speculate that pre-micronemes and pre-rhoptries may bud from the novel and yet-uncharacterized vesicles that are visible above the nuclei of *Tg*Vps9-depleted mutants. As discussed above, *Tg*Vps9 displays *in vitro* GEF towards human Rab5A, comparable to that of Rabex5^18^,^19^,^20^,^21^,^22^,^23^. It follows then that some phenotypic traits of *Tg*Vps9-deficient mutants resemble those previously reported for *Tg*Rab5A protein after its encoding gene has been disrupted^31^. As both *Tg*Rab5A and Vps11 probably interact through the CORVET-tethering complexes^13^, this could explain their phenotypic similarities with those observed for *iKOTgVps9* mutants described here.

Protein trafficking that relies on VPS9 has been described in several other eukaryotic cells such as yeast, which has three Vps9-domain containing proteins, Vps9, Muk1 and Vrl1, all exhibiting GEF activity towards Rab5 paralogs^32^. Mammalian cells contain at least nine Vps9 domain-containing proteins fulfilling diverse functions including regulation of protein transport, endocytosis and signaling pathways^22^. Additionally, it has been reported that Vps9 domains also interact with retromer complex and phosphatidylinositol 3-phosphate (PI3P) to promote the enrichment of PI3P lipids at the endosomes^33^. Knowing that *Tg*SORTLR^10^ and the retromer machinery^7^ in *T. gondii* share similarities with those of *Tg*Vps9, the latter may participate in regulation of retromer and endosomal lipid content. However, *Tg*Vps9 is not associated with the Golgi apparatus like *Tg*SORTLR, suggesting that this parasite Rab5 GEF is likely involved in anterograde transport and secretory organelle formation, rather than protein recycling in *T. gondii*.

In conclusion, loss of *Tg*Vps9 inhibits rhoptry protein processing/maturation, impairs secretory organelle biogenesis and secretion, leading to an inability of *Tg*Vps9-deficient mutants to invade host cells and to achieve multiple rounds of invasion, proliferation and reinvasion of host cells. However, there is still a missing link between protein processing, maturation, vesicular traffic and secretory organelle formation. Further investigation of *Tg*Vps9 functions that will define how different parasite *Tg*Rab proteins are precisely regulated by its GEF activity could provide this missing link.

## Methods

### Parasite culture

We used *T. gondii* tachyzoites of RH strain for CRISPR/Cas9 knockout experiment, RHΔ*Ku80*^34^ for the knock in of *Tg*Vps9 gene (TGME49_230140) and RHΔ*Ku80*TATi for inducible knockout (iKO) strain^35^,^36^ that were grown using Human Foreskin Fibroblast (HFF) cells from ATCC (USA) as described^7^. The iKO*Tg*Vps9 mutants were cultured in the presence of 1.5 μg/ml anhydrotetracycline (ATc).

### Production of recombinant *Tg*Vps9, Rabex5, and Rab5 proteins

Recombinant protein of the catalytic core of *Tg*Vps9 from aa_849_ to aa_1134_ was generated using a modified pET19 plasmid that expresses His-tag protein with a TEV cleavage site using the following primers: forward (Recomb-Vps9a.d4_F) CCGGCATATGGCGTCTTCTGCCTCTTTTTCTGCC and reverse (Recomb-Vps9a.d4_R) CCGGGGATCCTTAGCGTTCGCGTTCGCGGTCGTATTC. Human recombinant Rabex5 from aa_132_ to aa_397_ and full length Rab5a with a C-terminal CVIL mutation were prepared as previously described^37^. The recombinant *Tg*Vps9 was expressed in BL21 Codon Plus (DE3)-RIPL, and cell pellet was resuspended in buffer A (50 mM HEPES pH 8, 300 mM NaCl, 1 mM Tris (2-carboxyethyl) phosphine (TCEP), 20 mM imidazole pH 8), sonicated, and centrifuged at 20,000 rpm for 1 hour at 4 °C. Cell lysate was incubated with 2 ml agarose Ni-NTA beads, washed with buffer A and subsequently buffer B (50 mM HEPES pH 8, 300 mM NaCl, 1 mM TCEP, 30 mM imidazole pH 8). Bound protein was eluted using buffer C (50 mM HEPES pH 8, 300 mM NaCl, 1 mM TCEP, 500 mM imidazole pH 8). His-tags were removed by TEV protease cleavage during overnight dialysis in Buffer A without TCEP and imidazole. In order to remove the His-tagged TEV protease, dialyzed protein solution was incubated with Ni-NTA beads again before the GEF assays were performed.

### GEF assay

GEF activities were analyzed by intrinsic tryptophan fluorescence measurements showing fluorescence changes due to the conformational change from GDP to GTP state^19^,^20^. Rab5 fluorescence was excited at 297 nm and emission signals were detected at 340 nm. The fluorescence was recorded using a Cary Eclipse fluorescence spectrophotometer (Agilent Technologies). 10 μM of 5′-Guanylyl imidodiphosphate (GppNHp), non-hydrolysable analog of GTP, or 10 μM GDP were added to 1 μM of Rab5 GDP in GEF assay buffer (25 mM HEPES pH 7.5, 200 mM NaCl, 1 mM MgCl_2_, 2 mM dithioerythritol (DTE)), subsequently 200 nM or 400 nM of *Tg*Vps9 or 200 nM of Rabex5 were added.

### Generation of stable transgenic strains

Endogenous gene tagging methodology using pLIC-HA-DHFR plasmid^34^ was used to generate *Tg*Vps9-HA knock in parasites. DNA of *Tg*Vps9 was cloned in this plasmid using the following primers: forward (F-KI_Vps9) TACTTCCAATCCAATTTAATGCCCCTGCTTGCCCCTCGCCT and the reverse (R-KI_Vps9) TCCTCCACTTCCAATTTTAGCTTTCCTGTCACTATGTTTCGCGTCCG. To obtain iKO*Tg*Vps9 mutants, we used pG13-D-T7S4 plasmid^36^ in which a 2-kb DNA containing the promoter sequence was cloned using the following primers: forward (iKO*Tg*Vps9-5′_F) CCGGCATATGCTTCTAACGGCACCACTTAAGGTGC and reverse (iKO*Tg*Vps9-5′_R) CCGGCATA TGTGCGCCTTCTCGTGTCGTCTTG; and another 2-kb DNA containing the coding sequence of *Tg*Vps9 gene using the following primers: forward (iKO*Tg*Vps9-3′_F) CCGGTGATCAATGTACCCAT ACGATGTTCCAGATTACGCTCGTCACGGGGAAGAAGACCAGCACGTC and reverse (iKO*Tg*Vps9-3′_R) CCGGCCTAGGGGGAGAAGAGGAGACAGAAACATCTCGACTACGACC with the HA-tag sequence underlined in the forward primers inserted at the N-terminus of *Tg*Vps9 protein, right after the initiation ATG codon. 1 × 10^7^ parasites were transfected with 50 μg of linearized plasmid and selected with 2 μM of pyrimethamine. The emerging pyrimethamine-resistant population was cloned by limiting dilution. The clones were checked for plasmid integration by PCR using genomic DNA and two primers: forward (named A or Test_iKOVPS9.F2) ATTACAGCCAGCAGTGGCCAACCGAAT and reverse (named B, DHFR-int. R) GGCGTTGAATCTCTTGCCGACTGTGGAGAGGGAAGTCC.

### Immunofluorescence microscopy

Confocal microscopy was performed as described previously^10^,^38^. Briefly, intracellular parasites within HFF cells on 24-well coverslips were fixed by 4% paraformaldehyde for 10–15 min at room temperature. Fixed cells were permeabilized with 0.2% Triton X-100 and blocked with 5% fetal bovine serum (FBS). These cells were incubated with primary antibodies for 1 h at 37 °C and sequentially stained with secondary antibodies conjugated with Alexa 488, 594 or 647 in addition to DAPI for 30–45 min at 37 °C. Stained cells were mounted with Mowiol. All images were captured by a confocal microscope LSM780 or 880 (Carl Zeiss). Image processing was performed by open-source Fiji software.

### Western blots

Intracellular iKO*Tg*Vps9 mutants or parental parasites incubated with ATc or not for 48 h were scrapped and washed twice with PBS. The intracellular parasites were pelleted before suspension by Laemmli buffer (62.5 mM Tris-HCl pH 6.8; 2% SDS; 100 mM DTT; 10% sucrose) and boiled for SDS-PAGE. 2 × 10^6^ parasites were fractionated on 10% acrylamide gels, which were transferred to nitrocellulose membranes as previously described^39^. Immunoblot was performed using several anti-MIC and ROP antibodies in TNT buffer (100 mM Tris-HCl pH 7.6; 150 mM NaCl; 0.1% Tween20). All membranes were stained with antibodies specific to the glycolytic enzyme anti-ENO2^40^ as a loading control after stripping antibody.

### Electron microscopy

For transmission electron microscopy, cells were fixed in 2.5% glutaraldehyde prepared in 0.1 M cacodylate buffer and post-fixed in 1% osmium tetroxide in the same buffer. After acetonitrile dehydration, the pellet was embedded in Epon. Ultrathin sections (90 nm) were cut using a Leica UC7 ultramicrotome and collected on 150 mesh hexagonal barred copper grids. After staining with 2% uranyl acetate prepared in 50% ethanol and incubation with a lead citrate solution, sections were observed on a Hitachi H-600 transmission electron microscope at 75 kV accelerating voltage.

### Invasion and plaque assays

Wild type RHΔ*Ku80*TATi strain and iKO*Tg*Vps9 parasites were incubated under ATc condition for 48 h and mechanically lysed by passage through a syringe. 1 × 10^5^ parasites were inoculated to HFF cells and incubated for 1 h at 37 °C. After infection for 1 hour, extracellular parasites were washed out with PBS and used to infect HFF cells before growing for 24 h at 37 °C. These infected cells were fixed by PFA and sequentially stained by GAP45 antibody with DAPI and counted by Axioimager Z1 (Carl Zeiss). Host cell invasion values were calculated using the ratio of intracellular parasite/host nucleus numbers as described^41^. For plaque assays, 400 freshly lysed parasites were used to infect HFF cells followed by incubation for 7 days with or without ATc. These cells were fixed by ethanol and stained by crystal violet.

## Additional Information

**How to cite this article**: Sakura, T. *et al*. A Critical Role for *Toxoplasma gondii* Vacuolar Protein Sorting VPS9 in Secretory Organelle Biogenesis and Host Infection. *Sci. Rep.* **6**, 38842; doi: 10.1038/srep38842 (2016).

**Publisher's note:** Springer Nature remains neutral with regard to jurisdictional claims in published maps and institutional affiliations.

## Supplementary Material

Supplementary Information
