# Supplementary material for: A Critical Role for Toxoplasma gondii Vacuolar Protein Sorting VPS9 in Secretory Organelle Biogenesis and Host Infection
Source: Sci Rep. 2016 Dec 14;6:38842. doi: 10.1038/srep38842 (PMC5155228; doi:10.1038/srep38842)
Supplement: Supplementary Information [file srep38842-s1.pdf]

SUPPLEMENTARY INFORMATION

A Critical Role for *Toxoplasma gondii* Vacuolar Protein Sorting VPS9 in Secretory Organelle Biogenesis and Host Infection

Takaya Sakura, Fabien Sindikubwabo, Lena K. Oesterlin, Hugo Bousquet, Christian Slomianny, Mohamed-Ali Hakimi, Gordon Langsley and Stanislas Tomavo

Supplementary Figure S1: Characterization of *T. gondii* vacuolar protein sorting VPS9

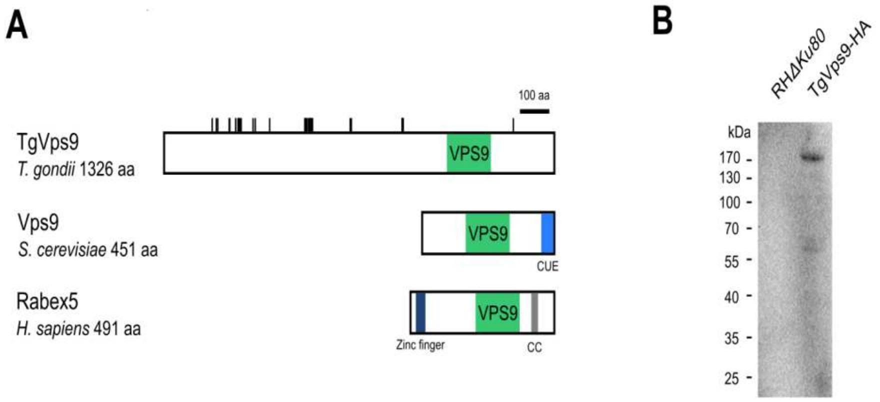

(A) Schematic representation of *Tg*Vps9 and comparison to Vps9 from *S. cerevisiae* and Rabex5 from *H. sapiens*. The yeast CUE domain regulates Vps9 function as ubiquitin binding domain in the endocytic pathway. The human N-terminal zinc finger sequence of Rabex5 promotes intramolecular ubiquitination. CC; coiled coil domain. No CUE, zinc finger or other domain has been identified in *Tg*Vps9 except the characteristic Vps9 domain. (B) Immunoblot analysis of HA-tagged *Tg*Vps9 obtained after gene knock-in strategy. Total protein extract of clonal knocked-in parasites was probed with specific anti-HA antibodies. The parental RHΔKu80 used as strain background and a negative control for the gene knock-in experiment showed no HA positive protein. The aberrant mobility of HA-tagged *Tg*Vps9 detected in transgenic parasites is likely due to its N-terminus being heavily

phosphorylated with the individual sites indicated as black bars in panel A. Short vertical lines indicate phosphorylation sites at corresponding positions.

Supplementary Figure S2: Ulstructural images showing different classical and other parasite-specific organelles in conditional *Tg*Vps9 knock out mutants

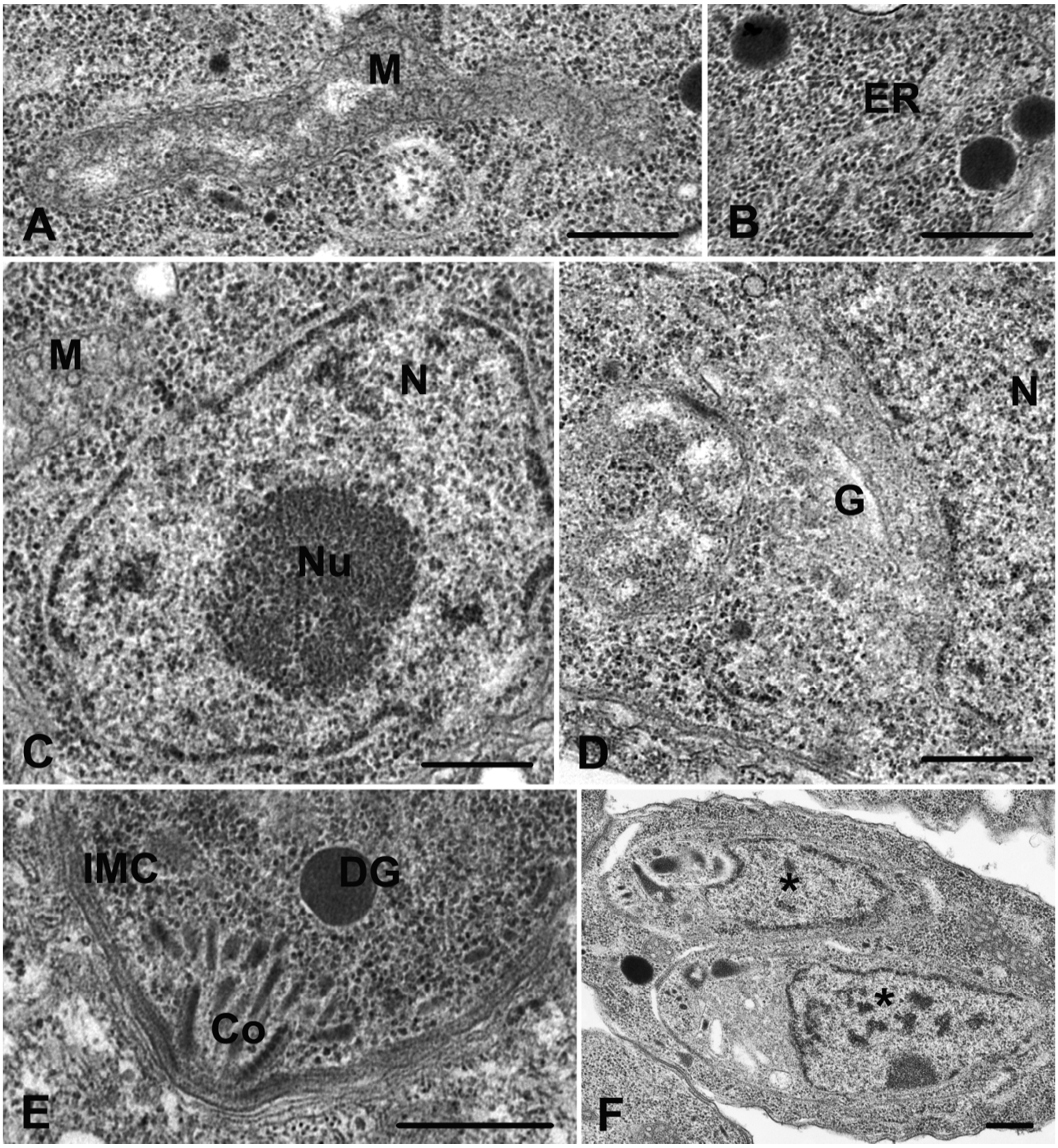

Ultrastructural images of sections representing *Tg*Vps9-depleted mutants after 48h of ATc

treatment were shown with the presence of morphologically intact mitochondria (panel A), endoplasmic reticulum (panel B), nucleus with typical nucleoli (panel C), Golgi apparatus (G), inner membrane complex (panel E). Panel F shows iKO*Tg*Vps9-deficient mutants, which appear to undergo normal endodyogeny with two daughters forming within the mother cell. (*****) indicates the nucleus in each daughter parasite; M, mitochondrion; ER, endoplasmic reticulum; N, nucleus; Nu, nucleoli; G, Golgi apparatus; IMC; inner membrane complex; DG, dense granules; Co, conoid. Scale bar indicates 500 nm.
